# Daily activities and self-esteem among university students with and without ADHD

**DOI:** 10.3389/fpsyt.2025.1622354

**Published:** 2025-08-26

**Authors:** Eryn H. Turner, Seth C. Harty

**Affiliations:** School of Psychology, Speech and Hearing, University of Canterbury, Christchurch, New Zealand

**Keywords:** ADHD, young adults, university students, self-esteem, daily activities

## Abstract

**Introduction:**

A diagnosis of ADHD has been repeatedly associated with low self-esteem in university students. Low self-esteem is associated with a range of negative outcomes including poor social function, increased psychopathology, and low academic self-efficacy. This study examined associations between ADHD, self-esteem, and daily activities in university students.

**Methods:**

A total of n =125 university students from New Zealand (50 meeting diagnostic criteria for ADHD) completed a survey measuring ADHD symptoms, self-esteem, and variables associated with self-esteem as well as a seven-day ecological momentary assessment survey measuring momentary self-esteem, mood, and current activity.

**Results:**

ADHD was associated with a range of negative outcomes including low self-esteem, low general and academic self-efficacy, and high levels of mental distress. Global self-esteem was associated with momentary self-esteem. Differences between students with and without ADHD were observed both in momentary self-esteem ratings and the frequencies of endorsed activities. In both groups, decreased momentary self-esteem was associated with being alone and procrastinating. For other activities, a differential pattern of momentary self-esteem increases was observed across groups.

**Discussion:**

This study provides support for the view that self-esteem is best conceptualised as both a stable trait and a state, fluctuating in response to daily events. Notably, this study also provides evidence that engagement in specific activities is associated with dynamic elevations in self-esteem among university students meeting diagnostic criteria for ADHD.

## Introduction

Attention-deficit/hyperactivity disorder (ADHD) is a neurodevelopmental disorder that has been shown to persist across the lifespan (1) with a worldwide prevalence of 5.9% in youth and 2.5% in adulthood (2). Characterised by symptoms of inattention, hyperactivity, and impulsivity, the diagnosis is associated with a range of negative outcomes including impairments in social, emotional, academic, health, and functional domains (1, 3, 4). Among university-aged students, research shows that, relative to non-ADHD university students, individuals diagnosed with ADHD have higher levels of emotional dysregulation, reduced social skills, and higher rates of psychological distress (5, 6). Additionally, these students exhibit lower academic performance (7) and academic coping skills (6), greater procrastination, and lower self-esteem (5).

Self-esteem can be broadly defined as an individual’s subjective evaluation of themselves (8); it refers to an individual’s conceptualization of who they are and their value as a person (9). Self-esteem is distinct from self-concept, which refers to an individual’s understanding of themselves including both their qualities and appearance (10), and self-worth, which can be understood as an individual’s sense of whether they meet their own standards regarding their personality and achievements (11). Self-esteem can be examined globally, providing a summative conceptualization of an individual’s self-evaluation or it can be examined across specific domains (e.g. academic self-esteem) (12). As such, global self-esteem can be conceptualized as the of sum of domain-specific self-esteem conceptualizations. Global self-esteem has been shown to be correlated with domain specific self-esteem, suggesting a general consistency within the construct (12).

Self-esteem is not necessarily reflective of an individual’s objective skills and traits (8). However, high self-esteem has been associated with self-worth and self-acceptance (8) and high rates of global self-esteem have been linked to positive outcomes in social, occupational, and health domains (13). Conversely, lower global self-esteem has been associated with a wide range of difficulties in inter- (14) and intrapersonal function (15). In university students, low self-esteem has been shown to be predictive of difficulties in social function in general (16) as well as among individuals diagnosed with ADHD (17). Additionally, low self-esteem in the first two years of university has been found to predict low academic self-efficacy in subsequent years (18).

Individuals with a diagnosis of ADHD have been found to have lower self-esteem compared to their non-ADHD peers in both adolescence (19) and adulthood (20). A recent meta-analysis of 127 studies revealed that when compared to individuals not diagnosed with ADHD, ADHD individuals had worse self-esteem and self-esteem related outcomes (3). Additionally, dimensional comparisons have revealed that individuals displaying more severe ADHD symptoms have lower self-esteem relative to those with milder symptoms (21). These relationships have also been observed in university students with studies finding that students with ADHD have lower self-esteem than students without ADHD and that self-esteem is negatively correlated with ADHD symptomatology (17, 22).

Low self-esteem among individuals with ADHD has been proposed to be an outcome of ADHD symptomatology where enduring negative consequences associated with ADHD symptoms negatively affect self-esteem (23). Such theoretical conceptualizations have been supported by previous research which has shown that individuals with ADHD tend to have poorer academic performance (24), experience more peer rejection (25), and experience more negative parent-child interactions in childhood (26), all of which have been associated with low self-esteem in individuals with ADHD (20). In addition to the relation between low self-esteem and the sequalae of consequences as a function of ADHD symptoms, there appears to be a direct effect of symptoms on self-esteem, as recent studies have shown that adolescents meeting criteria for ADHD describe their experiences of the core symptoms of ADHD negatively (24, 27) with qualitative studies indicating that adolescents with ADHD feel different and marginalized by these experiences (24, 25). While the cognitive and neuropsychological mechanisms supporting the association between ADHD and self-esteem are underexamined, a recent study found that across subtypes, working memory, which has repeatedly been associated with ADHD diagnosis and symptom severity (26), was negatively correlated with self-esteem (28). Lastly, as the initiation of treatment for ADHD symptoms has been shown to result in significant improvement in self-esteem (3), it appears that there is a robust link between ADHD symptoms and consequences to self-esteem.

Self-esteem has been shown to be a stable construct, such that individuals who experience low self-esteem relative to their peers at one point in their life are more likely to have low self-esteem relative to their peers at other points in their life (8). Traditionally, the measurement of self-esteem has been measured using survey-based methodologies and has often been assessed at singular time points. These singular assessments reflect global self-esteem (29); however, self-esteem has been shown to be both stable over time as well as fluctuate across moments (30, 31). Such discrepancies have resulted in an ongoing debate regarding whether self-esteem is best categorised along state or trait dimensions (32). Some researchers argue that the stability of global self-esteem over the lifespan indicates that self-esteem is best conceptualised as a trait (33) while others emphasise the differential influence of situational factors on self-esteem (34, 35). Results of a study examining self-esteem using a latent variable trait-state model indicated that self-esteem contains both trait and state components, indicating that there is utility in examining self-esteem as both fixed and fluctuating (32). Research that has examined state changes in self-esteem through the assessment of multiple daily ratings of self-esteem has found that higher self-esteem variability has been associated with negative outcomes such as increased internalising symptoms (36), maladaptive reactions to negative feedback (35), and social anxiety and avoidance (37). It has been proposed that such fluctuations in self-esteem are a result of recently experienced self-relevant experiences (35) and research has linked negative interpersonal events, daily challenges, negative generalisation, and depressive symptoms to increased self-esteem variability in young people (35, 36, 38). Conversely, a study by Sowislo et al. (39) found no effect of self-esteem contingency (defined as fluctuations in self-esteem based on self-relevant events) and only a small effect of self-esteem instability (defined as short-term variability in self-esteem) on depressive symptoms in adults. In a study of daily self-esteem fluctuations in young adults, DeHart & Pelham (40) found that individuals with reported low trait self-esteem were particularly vulnerable for state implicit self-esteem fluctuations following negative events. This suggests that trait self-esteem may act as a buffer to reduce the effects of negative events on state self-esteem fluctuations. Overall, findings indicate that while self-esteem may remain generally stable across time, it varies within the day in response to contextual fluctuations which may differentially influence individuals with low and high, levels of global self-esteem. These findings have specific relevance to individuals diagnosed with ADHD. In addition to reporting low self-esteem (20), individuals diagnosed with ADHD experience significantly more negative daily life events compared to their non-ADHD peers (41). Further, among individuals diagnosed with a psychiatric disorder, lower self-esteem has been identified as a risk and perpetuating factor (42, 43). As such, understanding the influence of the construct among individuals with ADHD warrants continued consideration.

To our knowledge, no research to date has specifically examined daily self-esteem patterns and consequences among individuals diagnosed with ADHD. Ecological momentary assessment (EMA) is a research assessment protocol that allows for the repeated sampling of behaviours occurring in real time, conducted in the natural environment (44). The advantage of EMA over traditional methods is that it allows for the measurement of variables both repeatedly and in real time. The use of EMA methodologies has been successfully used in studies with children, adolescents, and adults diagnosed with ADHD (45), however, none have been used to specifically assess self-esteem as an outcome variable.

The present study aimed to examine self-esteem across seven days in young adult university students with and without ADHD. In line with previous research, it was hypothesised that when compared to their non-ADHD peers, individuals meeting criteria for ADHD would report lower rates of global self-esteem and that these differences would be seen across daily assessments. As individuals with ADHD have been found to experience more negative daily life events, it was also hypothesised that individuals meeting criteria for ADHD would report experiencing a greater number of potentially stress-inducing activities (e.g. procrastinating) and that these activities would be associated with relatively greater changes in reported self-esteem.

## Materials and methods

### Participants

A total of n=185 late adolescent and young adult university students residing in New Zealand signed up to participate in this study. Of those, 21 did not meet study criteria and 39 did not engage in the study after providing consent. The final sample of n = 125 participants included 18 males, 106 females, and one participant who reported being intersex. Age ranged from 17 to 21 (M=18.2, SD = 0.85). Participants were initially recruited through their participation in another study (20.8%).To increase sample size, additional participants were recruited through a first-year psychology course (79.2%). All participants were students at a regional university in New Zealand.

### Materials

ADHD symptom severity was measured using the Adult ADHD Self Report Scale (ASRS; 46). The ASRS is an 18-item self-report scales that measures ADHD symptoms on a 5-point Likert scales with 1 being “never” and 5 being “very often”. This measure is frequently used in samples of individuals with ADHD, and in this sample, this scale had excellent internal consistency (α = 0.92). Item responses were averaged to create mean scores for total ADHD.

Global self-esteem was measured using the Rosenberg Self-Esteem Scale (RES; 32). The RES is a 10-item self-report scale that measures self-esteem (e.g. *I take a positive attitude toward myself*) on a 4-point Likert scale with 1 being “strongly agree” and 4 being “strongly disagree” (47). Items are averaged to create an overall mean score. In the present sample, the RES demonstrated high internal consistency (α = .89).

The New General Self-Efficacy Scale (NGSES (48) is an 8-item scale measuring the extent to which individuals believe they can achieve their goals. Items (e.g. *Even when things are tough, I can perform quite well*) are assessed using a 5-point Likert scale with 1 being “strongly disagree” and 5 being “strongly agree”. The NGSES scale has been found to have high reliability and validity (48) and was found to have high internal consistency in the present sample (α =.89).

The belief individuals have in their ability to achieve academic goals was measured using the 5-item General Academic Self-Efficacy Scale (GASES (49). Items (e.g. *I generally manage to solve difficult academic problems if I try hard enough*) are measured on a 5-point Likert scale with 1 being “strongly disagree” and 5 being “strongly agree” and averaged to create a single measure of academic self-efficacy. The GASES has been found to be both reliable and valid (49) and in the present sample, the GASES demonstrated high internal consistency (α = .81).

The Depression, Anxiety, and Stress Scale (DASS-21) is a 21-item scale measuring depression, anxiety, and stress on separate subscales using a 4-point Likert scale with 0 being “did not apply to me at all” and 3 being “applied to me very much, or most of the time” (50). In the present sample, the DASS-21 had high-acceptable internal consistency with α = .87 for the depression subscale, α = .80 for the anxiety subscale, and α = .80 for the stress subscale.

Emotional reactivity was measured using The Perth Emotional Reactivity Scale short form (PERS-S (51),). The PERS-S is an 18-item scale that measures emotional reactivity on a 5-point Likert scale with 1 being “very unlike me” and 5 being “very like me”. This scale consists of two nine item subscales measuring positive emotional reactivity (e.g. *I tend to get happy very easily*, α = .78) and negative emotional reactivity (e.g. *I tend to get upset very easily*, α = .86). For each subscale, items are averaged to create an overall mean score.

The Baruth Protective Factors Inventory (BPFI) is a 16-item scale measuring factors that contribute to resilience, such as social support, adaptive personality, and compensating experiences (52). A score on the BFPI serves as a unitary measure of protective factors across all the measured domains. The BFPI uses a 5-point Likert scale with 1 being “strongly agree” and 5 being “strongly disagree” (52). In the present study the BFPI has been found to have high internal consistency (α = .83).

The Smartphone Ecological Momentary Assessment (SEMA^3^) digital application was used to deliver the ecological momentary assessment survey. This application was developed by the Melbourne eResearch group (53) and allows researchers to send surveys to participants’ iOS or Android mobile devices to be answered in real time. The survey consisted of the following five questions:

1. “How are you feeling?”
  Participants selected relevant emotions from the following list: “content,” “happy,” “energetic,” “excited,” “neutral,” “bored,” “tired,” “anxious,” “scared,” “stressed,” “angry,” “irritated,” “jealous,” “sad,” “upset,” and “disappointed”.
2. “How intense is your feeling?” measured using a 10-point Likert scale from 1 (low intensity) to 10 (high intensity).
3. “Rate your current self-esteem,” measured using a 10-point Likert scale from 1 (very low) to 10 (very high).
4. “What are you doing?”
  “Participants were able to select activities from the following list: “spending time with family,” “spending time with friends,” “spending time alone,” “at university,” “doing homework or studying,” “working,” “volunteering,” “exercising,” “participating in an extracurricular activity,” “at a sports game or practice,” “attending a social event,” “relaxing,” “doing a hobby/something fun,” “using social media,” “getting organised for something (e.g. university, bed, socialising),” “procrastinating a task (e.g. homework, chores, getting ready),” “eating,” and “having an argument”.
5. The final question was: “Are your feelings related to what you’re currently doing?”. Participants were able to choose “yes,” “no,” or “not sure”.

### Procedure

Data was collected in between September 2023 and June 2024. At the start of this study, individuals provided informed consent, completed the questionnaire battery which was hosted on Qualtrics, and were given instructions to download the SEMA^3^ application onto their mobile device. Starting the following day, participants were sent notifications to their mobile device with instructions to complete the EMA questions. Participants received five notifications per day for seven consecutive days. Notifications were sent in the early morning (8am), late morning (11am), early afternoon (2pm), early evening (5pm), and late evening (8pm). Each survey was available for completion until the next time point notification was sent out. Upon completion of the study, participants were compensated with either course credit or a grocery voucher. The current study was reviewed and approved by the Human Ethics Committee at the University of Canterbury, Christchurch, New Zealand.

### Data analysis

Group membership (ADHD/non-ADHD) was determined following DSM-5-TR (54) guidelines requiring the presence of 5 or more symptoms of inattention and/or hyperactivity-impulsivity that have persisted for at least 6 months and reported as maladaptive and impairing. Symptoms were considered present if rated “often” or “very often”. Within the ADHD group, a Kruskal-Wallis test was conducted to assess possible subgroup differences in reported ADHD symptoms.

Pearson correlations and independent samples t-tests were conducted in order to examine the relations and group differences (ADHD/non-ADHD) between global self-esteem, total ADHD symptom severity, and the constructs measured in the questionnaire battery.

Assessment of EMA data involved the use of two-way mixed model ANOVAs to examine group differences in self-esteem across and within days. Overall mean EMA reported self-esteem and self-esteem variability, as assessed as overall mean self-esteem SD, was examined as a function of group status and in relation to reported global self-esteem and ADHD symptoms. Chi-square tests of independence were calculated to assess differences in activity engagement.

Independent samples t-tests were conducted to examine self-esteem as a function of activity engagement. Mean self-esteem ratings were compiled for each reported activity. Group comparisons were conducted for each activity. Activity self-esteem means were also compared against the overall self-esteem mean score for each group. All analyses were conducted using Jamovi.version 2.6.44.

## Results

As can be seen in **Table 1**, correlation analyses revealed significant negative correlations between ADHD symptomatology and self-esteem, self-efficacy, academic self-efficacy, and protective factors. ADHD symptoms were negatively correlated with ratings of depression, anxiety, stress, and negative emotional reactivity. Self-esteem was positively correlated with measures of academic and self-efficacy and positive emotional reactivity and negatively associated with depression, stress, anxiety, and negative emotional reactivity.

**Table 1:** Correlations between study variables.

Variable	1	2	3	4	5	6	7	8	9
1. ADHD Symptoms	–	–	–	–	–	–	–	–	–
2. Self-Esteem	-.61***	–	–	–	–	–	–	–	–
3. Self-Efficacy	-.57***	.72***	–	–	–	–	–	–	–
4. Academic Self-Efficacy	-.53***	.60***	.81***	–	–	–	–	–	–
5. Depression	.54***	-.73***	-.58***	-.48***	–	–	–	–	–
6. Anxiety	.56***	-.65***	-.56***	-.46***	.65***	–	–	–	–
7. Stress	.62***	-.61***	-.47***	-.44***	.64***	.68***	–	–	–
8. NER	.52***	-.53***	-.46***	-.41***	.57***	.54***	.65***	–	–
9 PER	-.07	.30***	.26**	.15	-.33***	-.18	-.12	-.11	–
10. Protective Factors	-.55***	.70***	.69***	.58***	-.66***	-.50***	-.54***	-.48***	.34***

NER, Negative emotional reactivity; PER, Positive emotional reactivity *p <.05, **p <.01, ***p <.001.

Based upon endorsement of ADHD diagnostic criteria, a total of 50 participants (40%) were assigned to the ADHD group and 75 participants (60%) were assigned to the non-ADHD group. Of those assigned to the ADHD group 21 (42%) had previously been formally assessed for ADHD, 10 (48%) of these had preexisting diagnoses and 11 (52%) were assessed as part of participation in a previous study. The remaining 29 (58%) endorsed symptoms meeting or exceeding the diagnostic ADHD symptom criteria. Within the ADHD group there was no difference in ADHD symptoms between participants who had previously received a diagnosis of ADHD (*M* = 68.8, *SD* = 8.39), participants who completed a clinical interview assessing them for ADHD as part of another study (*M* = 72.9, *SD* = 8.35), and participants who were assigned to the ADHD group based on their endorsement of symptoms meeting criteria for ADHD (*M* = 66.3, *SD* = 9.01), χ^2^ (2) = 5.10, *p* = .08. There was no difference between the ADHD group and the non-ADHD group in age (t = -1.69, p = .09), sex (χ2 = 1.10, p = .58), or gender (χ2 = 1.49, p = .48).

Group comparisons, as seen in **Table 2**, revealed that the ADHD group reported significantly lower levels of self-esteem, self-efficacy, academic self-efficacy, positive emotional reactivity and significantly higher depression, anxiety, stress, and negative emotional reactivity. The magnitude of effect in these differences was large (*d* ranging from 0.79 to 1.07) for all variables except positive emotional reactivity which was small.

**Table 2:** Group differences for measured variables.

Variable	Group	Mean	SD	t	d
Self-Esteem	Non-ADHD	18.16	4.26	5.10***	0.93
	ADHD	13.86	5.12		
Self-Efficacy	Non-ADHD	30.39	4.26	5.28***	0.97
	ADHD	25.84	5.31		
Academic Self-Efficacy	Non-ADHD	18.91	2.80	4.30***	0.79
	ADHD	16.16	4.32		
Depression	Non-ADHD	4.76	3.58	-5.15***	0.94
	ADHD	8.54	4.58		
Anxiety	Non-ADHD	5.23	3.72	-4.60***	0.84
	ADHD	8.64	4.50		
Stress	Non-ADHD	6.65	3.65	-5.82***	1.07
	ADHD	10.78	4.19		
NER	Non-ADHD	29.21	6.56	-5.11***	0.94
	ADHD	35.12	5.93		
PER	Non-ADHD	32.96	4.73	2.10*	0.39
	ADHD	31.00	5.57		
Protective Factors	Non-ADHD	64.27	7.59	5.44***	1.00
	ADHD	56.46	8.19		

NER, Negative emotional reactivity; PER, Positive emotional reactivity *p <.05, **p <.01, ***p <.001.

### Ecological momentary assessment

Engagement with the EMA protocol was high, with 96% of surveys completed. As depicted in **Table 3**, frequency analyses revealed that significantly fewer surveys were completed in the morning compared to the rest of the daily assessment periods (χ^2^ = 74.4, *p* <.001, Cramer’s V = 0.13) and fewer surveys were completed later in the assessment period (χ^2^ = 13.0, *p* = .043, Cramer’s V = 0.05).

**Table 3:** Unsubmitted surveys by time and day.

Time	% not submitted	Day	% not submitted
8am	9.14	1	2.24
11am	3.09	2	2.88
2pm	3.09	3	4.00
5pm	3.09	4	4.96
8pm	1.94	5	4.00
		6	5.28
		7	5.12

This table shows the percentage of survey responses not submitted at each time point (across all seven days) and on each day (across all time points that day).

The overall mean of momentary self-esteem was significantly positively correlated with reported global self-esteem (*r* = .61, *p* <.001) and academic self-efficacy (*r* = .43, *p* <.001) and negatively correlated with ADHD symptomatology (*r* = -.46, *p* <.001). The ADHD group had significantly lower overall mean momentary self-esteem compared to the non-ADHD group (t = 4.65, p <.001, d = 0.85). Both groups completed a similar number of surveys (*t* = 0.86, *p* = .390, *d* = 0.16) and although ADHD symptomatology was negatively correlated with the number of EMA surveys completed, such that higher ADHD symptomatology was associated with completing fewer surveys, this correlation was not significant (*r* = -.17, *p* = .06).

**Figure 1:**
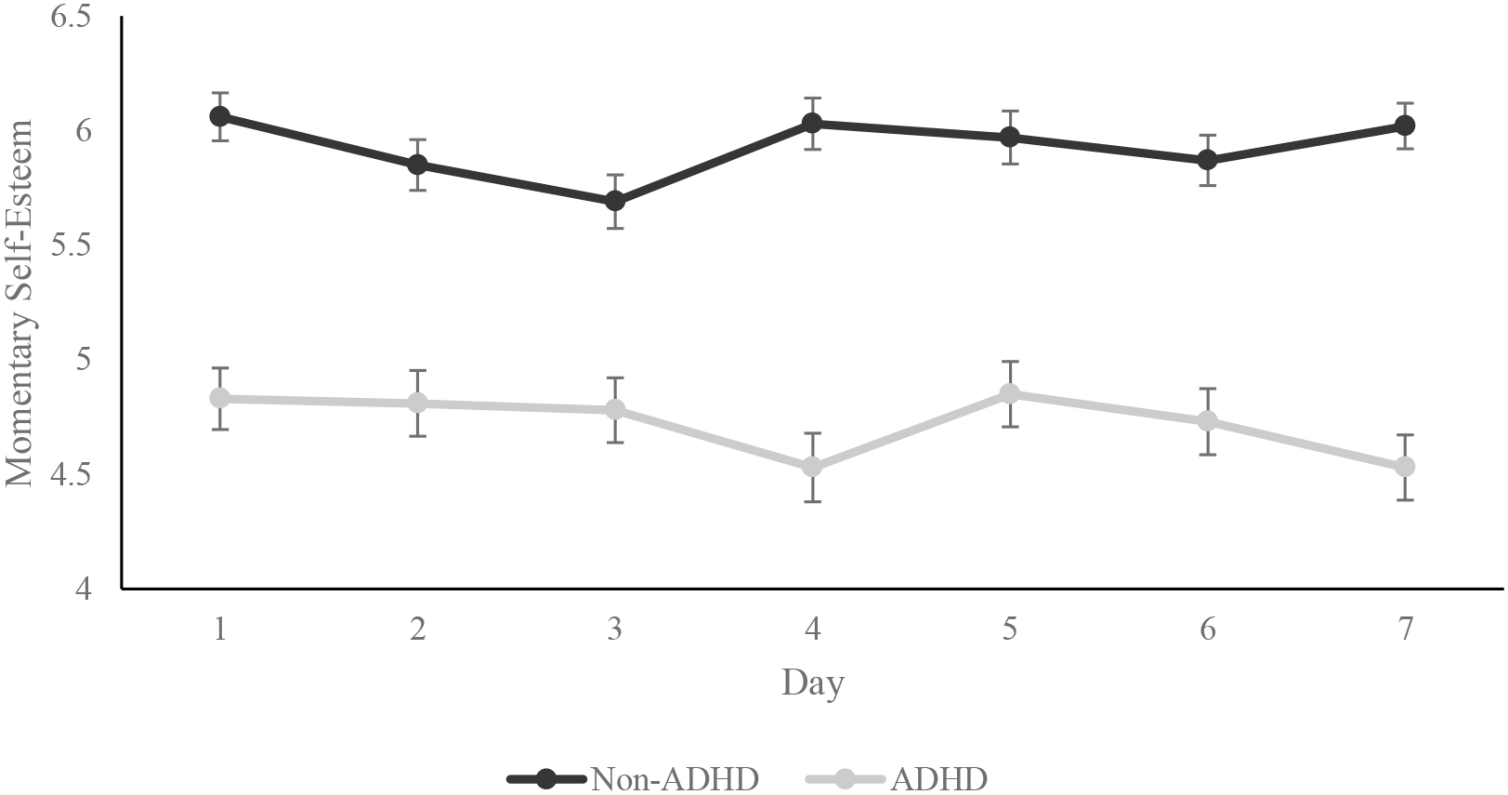
Group differences in mean momentary self-esteem across days. Error bars represent the standard error of the mean.

**Figure 1** presents group differences in momentary self-esteem across days. The overall model for the two-way mixed model ANOVA examining the effects of group and day was significant, F (13, 4183) = 26.87, p <.001. There was a significant main effect for group, F(1, 4183) = 336.70, p <.001, but not for day, (F(6, 4183) = 0.75, p = .61, or the group x day interaction, F(6, 4183) = 1.41, p = .21. These results demonstrate that self-esteem for individuals meeting criteria for ADHD is lower and stable across days.

**Figure 2:**
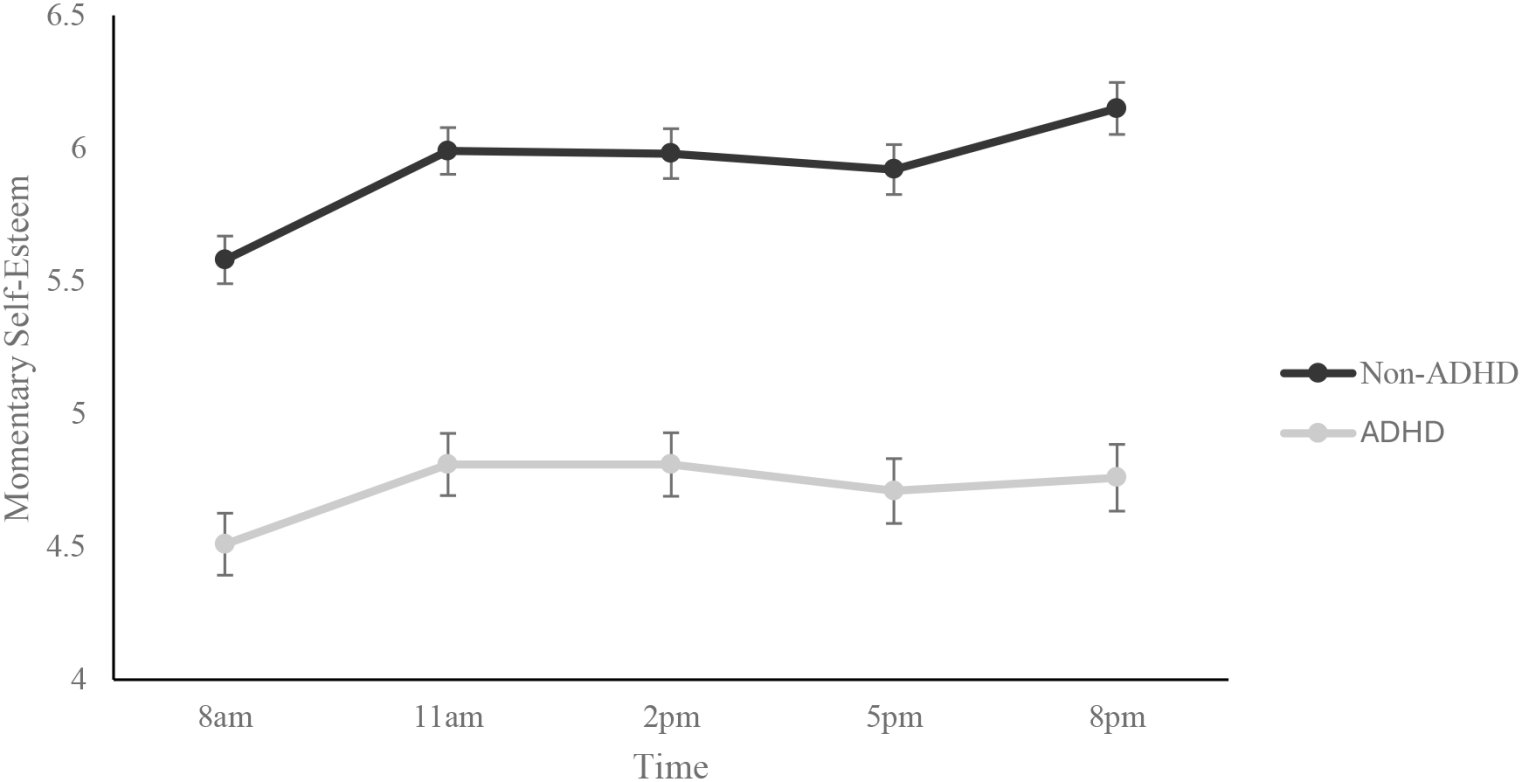
Mean momentary self-esteem across daily assessment intervals. Error bars represent the standard error of the mean.

**Figure 2** depicts mean self-esteem ratings across the daily assessment intervals. The overall model for the two-way mixed model ANOVA was significant, F(9, 4187) = 39.91, p <.001) with significant main effects for both group, F(1, 4187) = 336.76, *p* <.001 and time, F(4, 4187) = 4.22, *p* <.01. Non-ADHD individuals reported higher levels of self-esteem across all assessment intervals. *Post-hoc* analyses examining differences in assessment intervals revealed that self-esteem reported in the early morning (*M* = 5.19, *SD* = 2.07) was significantly lower when compared to late morning (*M* = 5.52, *SD* = 2.14), early afternoon (*M* = 5.50, *SD* = 2.23), and late evening (*M* = 5.62, *SD* = 2.36). The Group x Time interaction was not significant, F(4, 4187) = 0.51, *p* = .73 indicating that differences in daily self-esteem ratings were similar for both groups.

Variability in self-esteem, as measured by overall momentary self-esteem standard deviation was not significantly associated with ADHD symptoms (*r* = .12, *p* = .19) and was not found to significantly differ as a function of group status (*t* = -1.09, *p* = .28, *d* = 0.20).

### Activity engagement and momentary self-esteem

As shown in **Table 4**, examination of activity engagement frequencies revealed that studying, relaxing, and spending time alone and with friends were the activities most reported by both groups. While effect sizes were small, compared to the non-ADHD group, the ADHD group reported higher rates of procrastinating, being at university, working, getting organised, and lower rates of being with friends.

**Table 4:** EMA survey activity frequency split by group.

Activity	Non-ADHD	ADHD	Total %	χ^2^	Cramer’s V
Arguing	6 (0.2%)	8 (0.5%)	0.3	1.62	.02
At university	295 (11.8%)	243 (14.3%)	12.8	5.68*	.04
Being alone	508 (20.3%)	377 (22.2%)	21.1	2.13	.02
Being with family	182 (7.3%)	118 (6.9%)	7.1	0.169	.01
Being with friends	554 (22.2%)	328 (19.3%)	21.0	4.95*	.03
Doing hobbies	159 (6.4%)	121 (7.1%)	6.7	0.95	.02
Eating	176 (7.0%)	134 (7.9%)	7.4	1.06	.02
Exercising	90 (3.6%)	43 (2.5%)	3.2	3.77	.03
Extracurriculars	53 (2.1%)	26 (1.5%)	1.9	1.90	.02
Getting organised	287 (11.5%)	232 (13.7%)	12.4	4.43*	.03
Playing sports	31 (1.2%)	14 (0.8%)	1.1	1.65	.02
Procrastinating	185 (7.4%)	190 (11.2%)	8.9	17.8***	.07
Relaxing	508 (20.3%)	326 (19.2%)	19.9	0.81	.01
Socialising	71 (2.8%)	40 (2.4%)	2.6	0.93	.01
Studying	587 (23.4%)	358 (21.1%)	22.5	3.35	.03
Using social media	184 (7.4%)	101 (5.9%)	6.8	3.20	.03
Volunteering	13 (0.5%)	4 (0.2%)	0.4	2.03	.02
Working	87 (3.5%)	92 (5.4%)	4.3	9.29**	.05

Participant could select multiple activities when completing the surveys, so the percentages do not add to 100%, *p <.05, **p <.01, ***p <.001.

In order to understand the relation between activity engagement on self-esteem, mean momentary self-esteem scores were calculated for each endorsed activity. These scores were then compared against the calculated mean momentary self-esteem for all activities. These comparisons were done independently for each group. Differences were observed in the activities associated with increasing and decreasing momentary self-esteem across the two groups and are presented in **Tables 5** and **6**. For the ADHD group, being alone and procrastinating were associated with significantly lower momentary self-esteem, while being with friends, at university, working, socialising, or engaging in hobbies were associated with significantly higher momentary self-esteem. For the non-ADHD group, being alone, procrastinating, studying, and arguing were associated with significantly lower reported self-esteem while being with family, friends, relaxing, doing hobbies, and eating were associated with significantly higher momentary self-esteem.

**Table 5:** Significant differences in ADHD group momentary self-esteem by activity relative to overall ADHD group momentary self-esteem.

Activity	Mean	SD	t	d
At university	5.06	2.14	2.50*	0.16
Being alone	4.32	2.38	-3.27**	0.17
Being with friends	5.17	2.32	3.52***	0.19
Doing hobbies	5.76	2.49	4.60***	0.42
Procrastinating	3.90	2.04	-4.82***	0.35
Socialising	5.75	2.78	2.34*	0.37
Working	5.23	2.09	2.33*	0.24

*p <.05, **p <.01, ***p <.001.

**Table 6:** Significant differences in Non-ADHD group momentary self-esteem by activity relative to overall non-ADHD group momentary self-esteem.

Activity	Mean	SD	t	d
Arguing	2.32	1.93	-4.63**	1.89
Being alone	5.51	2.15	-4.72***	0.21
Being with family	6.80	2.15	5.23***	0.39
Being with friends	6.68	2.05	8.26***	0.35
Doing hobbies	6.84	1.98	5.60***	0.44
Eating	6.51	1.99	3.66***	0.28
Procrastinating	5.19	1.99	-5.24***	0.39
Relaxing	6.16	2.04	2.20*	0.10
Studying	5.75	2.00	-2.54*	0.11

*p <.05, **p <.01, ***p <.001.

## Discussion

This study sought to better understand the relation between self-esteem and ADHD symptomatology in a sample of young adult university students living in New Zealand. Low self-esteem, a feature frequently found in studies on individuals diagnosed with ADHD, has been associated with a number of negative outcomes and has been posited to be a contributing factor in the maintenance of psychiatric disorders. Among university students, high rates of ADHD symptoms and low self-esteem have been associated with factors, such as procrastination (5), that are directly relevant to success in academic contexts. To date, studies examining self-esteem outcomes in individuals diagnosed with ADHD have considered self-esteem as a global construct, reflective of general trait like processes, and often measured at one point in time. However, self-esteem fluctuations have been associated with increased negative life events and shown to be more prominent among individuals with low, compared to high, self-esteem (40). The current study measured self-esteem multiple times a day across seven days. To better understand the contextual factors that contribute to daily self-esteem ratings, this study recorded activity engagement at each self-esteem measurement. It was expected that compared to their non-ADHD peers, individuals meeting criteria for ADHD would report lower rates of global self-esteem and that these differences would be seen across daily assessments. It was also hypothesised that individuals meeting criteria for ADHD would report engaging in a greater number of potentially stress-inducing activities and that these events would be associated with changes in reported self-esteem.

Consistent with previous literature demonstrating that self-esteem is associated with wellbeing (13), global self-esteem measured at the start of this study was associated with higher self-and academic-efficacy and positive emotional reactivity and negatively associated with depression, anxiety, stress, and negative emotional reactivity. Self-esteem was negatively associated with ADHD symptomatology. Initial group comparisons revealed that, compared to their non-ADHD peers, those who met or exceeded the criteria for a diagnosis of ADHD reported significantly lower self-esteem, self-and academic-efficacy, positive emotional reactivity and higher depression, anxiety, stress, and negative emotional reactivity. These comparisons generated large effect sizes. These results support study hypotheses and are consistent with a large body of research linking ADHD to a range of negative outcomes including lower self-esteem (19), lower self-efficacy (21) and academic self-efficacy (55, 56), poor mental health (57), and poor emotion regulation (58).

Self-esteem measured by the EMA protocol was significantly associated with a gold standard measure of global self-esteem, the Rosenberg Self-Esteem Scale (59). Additionally, group differences seen in initial group comparisons of global self-esteem were consistently reflected in measured momentary self-esteem both within and across days. These findings support the validity of the assessment of self-esteem used in the EMA protocol and the view that self-esteem is stable and trait like (8). However, as the frequency graphs of this study reveal, self-esteem is best conceptualised both as a state as well as a trait (30) where self-esteem is generally stable with fluctuations likely manifesting as a function of contextual factors (60) and individual differences (30).

As individuals diagnosed with ADHD have been shown to exhibit greater response variability across multiple areas of assessment (61) and that self-esteem variability has been associated with low rated self-esteem (40), it was hypothesised that the individuals meeting symptom criteria for ADHD would demonstrate greater fluctuations in EMA assessed self-esteem variability. Interestingly, group differences were not seen in self-esteem fluctuations. It is possible that our measure of variability, overall mean standard deviation, although a frequently used measure of performance variability in studies examining individuals diagnosed with ADHD (61), was not nuanced enough and more specific parameters may be better suited to examine the nature of state self-esteem variability.

Previous research has established an association between activity engagement and self-esteem such that engagement in activities that are physical in nature (62) or valued (63) have been associated with increased self-esteem, while other activities, such as excessive social media use (64), have been associated with lower self-esteem. Individuals diagnosed with ADHD have been shown to experience more frequent daily negative events (41) and greater impairment in daily functions (65) compared to non-ADHD individuals. While Whalen et al. (66) found that ADHD severity was positively associated with time in non-productive activities, to our knowledge, no studies have examined frequency differences in daily activities between those with and without ADHD.

Similarities and differences were seen in reported activities over the seven days. Both groups reported frequently being alone, with friends, studying, and working, activities which generally comport with other studies examining daily activities in university students (67). Of the few group differences seen in activity engagement, the strongest effect was seen in frequency of procrastination.

While not part of the diagnostic framework, procrastination, the tendency to delay or an activity that has a deadline, has been associated with symptoms of ADHD in university students (68). Recently, Bodalski et al. (5) found that self-esteem partially explained the relation between ADHD symptoms and procrastination in a sample of undergraduate university students. While this study did not consider self-esteem as a predictor of outcomes such as procrastination, the lower self-esteem and increased occurrence of procrastination among individual meeting criteria for ADHD in this study support such proposed relations. However, it was surprising to see that although individuals meeting criteria for ADHD procrastinated more, the effect of procrastination on self-esteem was similar across the two groups.

Individuals meeting criteria for ADHD also reported more frequently getting organised relative to their non-ADHD peers. Given that difficulties in organizational tasks is one of the symptom criteria of the diagnosis, it is not surprising that university students with ADHD would endorse more time spent getting organised. Despite this, it was notable that self-esteem was not found to be lower getting organized when compared to overall reported self-esteem.

Interestingly, students with ADHD reported spending more time at university than their non-ADHD peers and this was associated with increased self-esteem for this group. It was found that university students diagnosed with ADHD reported greater environmental mastery relative to their non-ADHD peers (69), suggesting that this group of individuals may be particularly adept at managing their environment and effectively using available opportunities. University campuses often offer opportunities for social engagement and past research has shown that students who are engaged in extracurricular clubs and organizations benefit in their psychosocial development (70). As such, it may be that university students diagnosed with ADHD who are adept at managing social environments, benefit from university campus offerings and this is reflected in relative increases in self-esteem. Future studies may wish to explore how spending time on campus could be used as a mechanism to improve self-esteem and academic self-efficacy in students with ADHD.

Relatedly, given the consistent findings, supported by this study, that self-esteem is lower among individuals diagnosed with ADHD and that individuals reporting lower self-esteem are at risk for increased negative outcomes, social factors associated with improved self-esteem have clinical relevance for this population. For individuals reporting clinically high levels of ADHD symptoms, engaging in hobbies, and social activities such as spending time with friends, socialising, being at university, and working were particularly notable for reports of elevated self-esteem.

Engagement in hobbies (71) and social interactions (72, 73) have been associated with improved self-esteem and future studies may wish to examine these social factors as possible mechanisms for raising self-esteem and improving mental health outcomes in individuals diagnosed with ADHD.

There were some limitations to this study and results should be interpreted with caution. While all individuals in the ADHD group endorsed meeting symptom criteria for ADHD, not all individuals competed a formal assessment process. Future research may wish to examine the relations identified in this study in a sample of formally diagnosed individuals. Further, this study only considered total ADHD symptoms, and it is possible that the findings seen in this study differ as a function of symptom cluster. In their review of long-term outcomes, Harpin et al. (3) noted that self-esteem outcomes were worse for non-medicated ADHD individuals and indicated that engagemnt with evidence-based interventions for ADHD resulted in improved self-esteem. This study did not assess history or use of pharmacologic or behavioural treatments. Lastly, female individuals were overrepresented in this study and findings may not generalise to young adult males with ADHD.

In their systematic review of self-esteem among adults with ADHD, Pedersen et al. (20) noted that there was a lack of studies examining the repeated finding of lower self-esteem in this population. This study highlights both the stability and fluctuating nature of self-esteem and reveals that while global self-esteem is stable, self-esteem can vary across the day and is affected by the activities individuals choose to engage in. Although there were activities, such as procrastination and getting organised, that individuals meeting criteria for ADHD engaged in more frequently than their non-ADHD peers, effect size differences were such that contextual differences alone would not be enough to account for stable low self-esteem. Psychiatric history has been shown to predict low self-esteem (74), and it is possible that the low self-esteem seen in this study is, in part, a function of enduring complications with difficulties in attention, hyperactivity, and impulsivity.

Self-esteem, while stable, is mutable (75). As such, consideration of activities, such as engagement with leisure, vocational, and social activities, that raise self-esteem merits further attention as possible intervention targets for this at-risk group. Clinicians may wish to focus on engaging university student clients with ADHD in activities such as spending time on campus that set them up for success and improve self-esteem. A recent study examining the challenges facing of university support services indicated a need for specialized interventions and support services tailored to meet the needs of students diagnosed with ADHD (76). The results of the current study, which identify specific situations and contexts that influence self-esteem, has the possibility to directly inform novel interventions targeting this vulnerable group of university students.

## Data Availability

The raw data supporting the conclusions of this article will be made available by the authors, without undue reservation.
